# Variation in Cone Beam Computed Tomography Utilization and Radiation Exposure Associated with Prostatic Artery Embolization on Two Separate Angiography Systems

**DOI:** 10.3390/jcm13237403

**Published:** 2024-12-05

**Authors:** Abin Sajan, Daniel W. Griepp, Ari J. Isaacson

**Affiliations:** 1 Department of Radiology, Columbia University Irving Medical Center, 622 West 168th Street, New York, NY 10032, USA; 2 Department of Neurosurgery, Henry Ford Providence Michigan State University, East Lansing, MI 48824, USA; daniel.griepp@hfhs.org; 3 IR Centers USA, Falls Church, VI 22043, USA; aisaacson@prostatecentersusa.com

**Keywords:** prostatic artery embolization, benign prostatic hyperplasia, cone beam computed tomography, radiation dose, angiography, imaging technique

## Abstract

**Background**: We aimed to compare cone beam computed tomography (CBCT) utilization and radiation exposure during prostatic artery embolization (PAE) procedures on two different angiography systems. **Methods**: PAEs performed by a single interventionalist between January 2018 and October 2020 on two multivendor angiography systems (AS1 and AS2) at a single center were retrospectively evaluated. Imaging techniques included CBCT acquisition when possible, predominantly from the distal aorta in AS1 and from the bilateral internal iliac arteries in AS2 (Discovery IGS 740, GE HealthCare, Chicago, IL). Baseline demographics, CBCT utilization and radiation doses, and total procedure radiation metrics for each group were collected and compared. **Results**: One hundred and twenty patients were analyzed in this study, with fifty-three patients (*n* = 25 in AS1, 28 in AS2) included as embolized bilaterally using CBCT. CBCT was acquired in 31% of the cases in AS1 and in 85% of the cases in AS2. Mean prostate volume was similar in both groups (103.0 mL vs. 130.1 mL, *p* = 0.23). There was no difference in fluoroscopy time, while the number of DSA series and CBCTs per case did differ in AS1 and AS2 (37.3 min vs. 32.1 min, *p* = 0.13, 19.8 vs. 8.0, *p* ≤ 0.001, 1.3 vs. 2.1 *p* ≤ 0.001). The mean total air kerma, total kerma area product and air kerma per CBCT were higher in AS1 compared to AS2 (2020.4 mGy vs. 490.3 mGy, *p* ≤ 0.001, 259.3 Gy*cm^2^ vs. 72.7 Gy*cm^2^, *p* ≤ 0.001 and 217.8 mGy vs. 45.8 mGy, *p* ≤ 0.001 respectively). To prevent confounding from a mean difference in body mass index, the data were adjusted using log outcome means, which corroborated the raw data findings. **Conclusions**: The mean procedural total kerma area product from AS1 was similar to that reported in other PAE studies, but it was substantially lower in AS2. The angiography system used has a significant impact on the ability to leverage CBCT and on overall patient and thus staff radiation exposure.

## 1. Introduction

Prostatic artery embolization (PAE) is a minimally invasive treatment for lower urinary tract symptoms secondary to benign prostatic hyperplasia (BPH). The worldwide prevalence of BPH is 210 million men, with an estimated prevalence of 50% in men over 50 in the United States [1,2]. PAE was originally performed for patients with hematuria, but PAE has increasingly become part of the standard of care with its recent inclusion in the American Urologic Association BPH guidelines [3,4]. PAE is considered a technically challenging procedure due to the potential difficulty identifying the prostatic arteries among the numerous and variable vessels in the pelvis, catheterizing small caliber, often tortuous prostatic arteries and preventing non-target embolization through the vast network of pelvic arterial anastomoses [5]. Because of these challenges, PAE procedure times can be lengthy, and patient and staff radiation exposures can be high, particularly for a less experienced operator [6].

The use of cone beam computed tomography (CBCT) during PAE has been described for multiple purposes. The initial publication on CBCT for PAE in 2013 described using the imaging modality to determine if microcatheter placement was correct prior to embolization to prevent non-target particle deposition [7]. Since then, additional reports have expanded on the use of CBCT for PAE to include catheter-directed CBCT angiography from either the distal aorta or internal iliac artery to determine the number of prostatic arteries, identify their origins, define the optimal microcatheter position for full prostate coverage while avoiding non-target branches, and generate a 3-D model for augmented fluoroscopic guidance during prostatic artery catheterization [8,9,10,11]. However, with the increased utilization of CBCT for PAE, concerns have arisen regarding the added radiation that additional acquisitions contribute to the total exposure [12]. A recent retrospective PAE study of 1476 patients demonstrated that the median effective radiation dose of PAE was 17.8 mSv [13]. There was no major radiation-related adverse events, and radiation dose correlated positively with body mass index (BMI) and fluoroscopy time.

At our institution, the PAE technique has evolved over time to attempt to improve success rates, increase procedural efficiency, and leverage the strengths of the available imaging equipment. After 5 years of performing PAE with one angiography system, a new angiography suite was installed and a new PAE workflow was implemented. This study sought to compare the newly implemented system with the prior, specifically in the setting of cone beam CT use, to assess if significant differences in radiation doses existed and how they contrast with the published literature on the topic.

## 2. Materials and Methods

All patients who underwent PAE in one of two angiography suites at a single academic medical center between 1 January 2018 and 13 October 2020 were retrospectively analyzed. This study was performed with institutional review board approval. However, since this study was a retrospective analysis, a waiver of informed consent was obtained. All PAE procedures were conducted by a single interventional radiologist, who had 5 years of experience performing PAE at the beginning of the study period. To meet inclusion criteria, only PAEs in which CBCT was used and bilateral (complete) embolization was achieved were included.

### 2.1. PAE Technique

CBCT was used when possible in both suites: The decision for or against the use of CBCT was not influenced by the angiography system used (AS1 vs. AS2). The decision to pursue transfemoral versus transradial access was non-random and primarily based on case-by-case differences in patient anatomy, with no influence by the angiography system used.

In angiography suite 1 (AS1), a floor-mounted, robotic-arm fluoroscope equipped with a 30 × 40 cm detector (Artis Zeego, Siemens Healthineers, Malvern, PA, USA) was used. A 5-French catheter was placed in the inferior abdominal aorta and a CBCT angiogram was obtained with the patient’s arms at their sides. In total, 50% concentration iohexol (Omnipaque 300, GE HealthCare, Chicago, IL, USA) at a rate of 4 mL per second for a total of 11 s was injected after a 5 s imaging delay. Additional imaging parameters included 6 s acquisition, frame rate: 66.67 fps, reconstructed 3D FOV 18.5 cm height × 24 cm diameter cylinder, and matrix size 512 × 512 × 512. These images were then reconstructed and used to identify the origins of the prostatic arteries bilaterally (Syngo Embolization Guidance, Siemens Healthineers). A 3-D rendered model was then overlaid on the live fluoroscopy images to guide the catheter to the prostatic artery. If the CBCT did not clearly define the origins of the prostatic arteries, digital subtraction angiography (DSA) was performed from each internal iliac artery to provide further information. Once the microcatheter was advanced into the prostatic artery, one or more DSA acquisitions was performed to identify the optimal positioning for embolization and potential arterial anastomoses to non-target organs. If necessary, additional CBCTs were acquired to clarify arterial anatomy. If needed, embolization coils were deployed in arterial anastomoses to prevent non-target particle deposition. In some cases, a post-embolization CBCT was acquired to evaluate the retention of contrast material within the prostate as a measure of the completeness of embolization.

Angiography suite 2 (AS2) included a mobile robotic angiographic system equipped with a 40 × 40 cm detector (Discovery IGS 740, GE HealthCare, Chicago, IL, USA). Instead of the abdominal aorta, a 5-French catheter was placed in the internal iliac artery. A CBCT angiogram was performed from this location with patients’ arms by their sides, also using an injection of 50% concentration iohexol (Omnipaque 300, GE HealthCare). The injection rate was 3 mL per second for a total of 10 s with a 3 s imaging delay. The acquisition time was 7 s and involved the following imaging parameters: 200-degrees rotation, frame rate: 50 fps, variable kVp and mAs to dynamically compensate for anatomical variations along the spin, reconstructed 3D FOV 24 cm height × 24 cm diameter cylinder, and matrix size 512 × 512 × 512. CBCT was then reconstructed and analyzed with embolization planning software (Embo ASSIST with Virtual Injection, GE HealthCare) to identify the locations of the origins of the prostatic arteries, possible non-target branches, and optimal embolization position. A 3-D rendered model of the arterial tree was then automatically overlaid on the fluoroscopic images to aid in catheter navigation. A digital zoom feature was used during augmented fluoroscopy, when possible, to avoid dose rate increase typically associated with conventional FOV-based magnification. Once the microcatheter had been advanced into the prostatic artery, DSA or CBCT images were acquired to confirm the optimal position for embolization and identify any collateral arteries leading to non-target organs. Again, coil embolization was performed within these collateral arteries if necessary. The same imaging technique was then utilized on the contralateral side, which included a second CBCT angiogram from the contralateral internal iliac artery.

### 2.2. Data Extraction

The data review was conducted using the electronic medical record and picture archiving and communication system. Information abstracted included age, body mass index (BMI), and volume of the prostate. Procedural data included the angiography suite used (AS1 or AS2), bilateral vs. unilateral success, access site (radial or femoral), whether coil embolization was performed, cumulative air kerma, total kerma area product, mean reference air kerma per CBCT, fluoroscopy time, the number of CBCTs and the number of DSA series.

### 2.3. Statistical Methods

Mean reference air kerma per CBCT, cumulative air kerma, and total kerma area product from procedures performed in each angiography suite were compared by using the exact Wilcoxon two-sample test with a continuity correction. Additionally, to control for the potential confounding effect of the BMI, an adjusted analysis was performed. For the adjusted analysis, to account for the outcomes not being normally distributed, a series of candidate Box–Cox transformations were evaluated using graphical methods and the Shapiro–Wilk test. For each outcome, the transformation yielding the largest Shapiro–Wilk statistic and the normal probability plot most closely approximating normality was the log transformation. An ordinary least-squared regression was used separately for log mean reference air kerma per CBCT, log cumulative air kerma, and log total kerma area product to model the relationship between each outcome and the angiography suite with the BMI included as a control variable, yielding adjusted (adjusting for the effect of the BMI) log outcome means. The difference in the adjusted log means for log mean reference air kerma per CBCT, log cumulative air kerma, and log total kerma area product was tested by the angiography suite. *p*-values < 0.05 for the test of difference in predicted means were considered evidence of a significant statistical difference. As a sensitivity analysis, multiple imputation was implemented to fill in missing values of the BMI, generating 50 complete case datasets using the Markov Chain Monte Carlo sampling methodology. To compare additional variables including baseline demographics and other procedural metrics, the Wilcoxon two-sample test with a continuity correction or Fisher’s exact test was employed where appropriate. *p*-values < 0.05 were considered significant. All analyses were performed using SAS, Version 9.4 (SAS Institute, Cary, NC, USA).

## 3. Results

**Figure 1:**
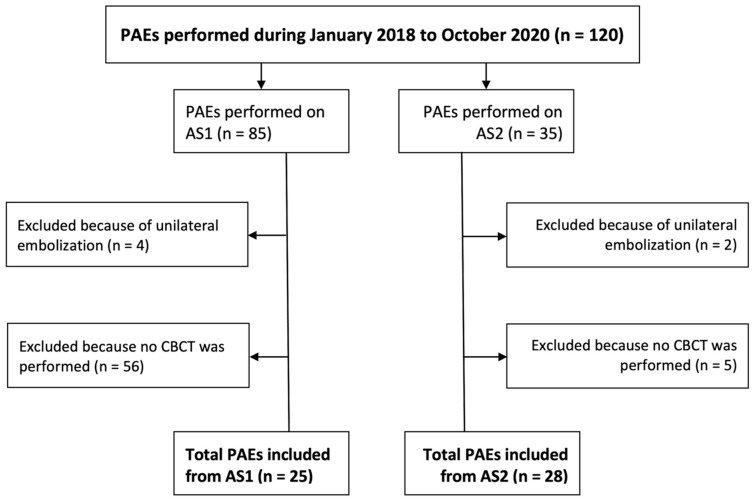
Study inclusion/exclusion flow chart.

During the study period, a total of 85 PAEs were performed in the first angiography system (AS1) and 35 were subsequently performed using the second angiography system (AS2). CBCT was acquired in 31% of the cases in AS1 and in 85% of the cases in AS2. After excluding procedures in which unilateral embolization was performed or no CBCT was acquired, 25 PAEs from AS1 and 28 from AS2 remained (Figure 1). Baseline patient demographics are provided in Table 1.

In AS1, 14 PAEs (56%) were performed from transfemoral arterial access and the remaining 11 (44%) from a transradial approach. In AS2, 21 PAEs (75%) were conducted from a transfemoral approach and the remaining 7 (25%) were transradial. Coils were deployed in 5/25 (20%) patients in AS1 and in 6/28 (21%) patients in AS2. The unadjusted mean reference air kerma per CBCT (217.8 vs. 45.8 mGy), cumulative air kerma (2020.4 vs. 490.3 mGy), mean total kerma area product (259.3 vs. 72.7 Gy*cm^2^), adjusted log mean reference air kerma per CBCT, log cumulative air kerma, and log total kerma area product were significantly higher for PAEs performed in AS1 compared to AS2 (*p* < 0.01). The results of the adjusted mean comparisons were corroborated by the sensitivity analysis using multiple imputation (*p* < 0.01 for all comparisons). A detailed comparison of the unadjusted data are shown in Table 2, and the adjusted, logarithmic data are shown in Table 3.

Additional analyses of procedural metrics demonstrated no significant difference in the mean fluoroscopy time between the two angiography suites (37.3 vs. 32.1 min, *p* = 0.13). However, the mean number of DSA series was higher in AS1 (19.8 DSAs in AS1 versus 8.0 DSAs in AS2, *p* < 0.001), while the mean number of CBCTs acquired was higher in AS2 (1.3 CBCTs in AS1 versus 2.1 CBCTs in AS2, *p* < 0.001) (Table 2).

## 4. Discussion

In the present study of CBCT utilization and radiation dose factors and outcomes amongst patients undergoing prostatic artery embolization, we found a significant increase in the use of CBCT and decrease in radiation exposure using the newly implemented angiography suite. While the fluoroscopy time was similar amongst both groups, indicating similar procedural complexity, the total air kerma and kerma area product were significantly lower in the AS2 group (2020.4 vs. 490.3 mGy and 295.3 vs. 72.7 Gy*cm^2^, respectively, *p* < 0.01). Moreover, the procedural mean total kerma area product from AS1, 259.3 Gy*cm^2^, was similar to the levels reported in multiple other PAE studies, while the mean total kerma area product from AS2, 72.7 Gy*cm^2^, was substantially less [14]. In addition to being significantly lower than what was observed in AS1, the mean reference air kerma per CBCT in AS2 was also substantially lower than the dose previously reported for “low-dose CBCT angiography” for PAE (45.8 mGy vs. 120.3 mGy) [15]. The data from this study display how total radiation dose during PAE and radiation mean reference air kerma per CBCT can vary significantly based on the angiography unit and procedural technique utilized. While the operator and the procedural learning curve are typically key parameters influencing radiation outcomes, examinations in both cohorts in this study were performed by the same experienced operator, allowing us to quantify the sole impact of the angiography unit and the technique used on radiation outcomes.

The importance of the association of the BMI to overall procedural radiation dose during PAE has been described [16,17]. Therefore, because the population in AS1 had a significantly higher BMI, an additional statistical analysis was necessary to determine if the overall increased radiation doses observed in AS1 were simply due to the difference in BMIs. To determine this, a statistical technique was employed in which log means adjusted for the BMI were compared, thus preventing potential confounding from the difference in the BMI between the two groups.

There is no ideal way to describe radiation dose due to CBCT [18]. In prior reports, it has been described in terms of total kerma area product or cumulative air kerma, sometimes called “skin entry dose” [15]. In this report, the decision was made to use cumulative air kerma, the rationale being that the total kerma area product is routinely published using different units (e.g., Gy*cm^2^, mGy*m^2^, uGy*m^2^). For most interventional radiologists, conversion between these units requires computer software, making a comparison of total kerma area product values from different papers difficult. Because cumulative air kerma is always reported in mGy or Gy, a comparison between different studies is simpler.

Limiting radiation exposure to the patient is an important objective during PAE because deterministic radiation injury during PAE has been reported [19]. Additionally, the risk of the stochastic effects of radiation after PAE have also been presented [14,20,21]. Finally, besides the patient, personnel in the angiography suite are also exposed to primarily scatter radiation and exposure can accumulate over time, increasing the concern of stochastic effects [22,23]. With personnel typically leaving the room during CBCTs, but remaining most often tableside for DSAs, the significant increase in CBCT use, the decrease in the number of DSA, and the reduction in patient radiation exposure demonstrated between AS1 and AS2 are likely associated with an even more significant decrease in occupational personnel radiation exposure between AS1 and AS2. A key benefit of the newer system is the ability to perform more CBCTs at lower doses, which reduces DSAs and decreases the overall radiation exposure.

The difference in total radiation dose that was observed between the two angiography suites can be partially explained by the difference in dose for the CBCT acquisition, but there are other factors. There was a significant difference in the mean number of DSA series acquired between the two sites, with more than double acquired at AS1. The reduced number of DSAs in AS2 was due to the improved distal anatomy visualization on internal iliac artery CBCTs compared to distal aorta CBCT and to the ability to use “augmented fluoroscopy” or 3D-rendered images from the internal iliac artery CBCT angiograms overlaid on fluoroscopic images for dynamic guidance in lieu of DSA images [21]. While this was also possible in AS1, because the CBCT angiogram was from the aorta and less selective, the images of the branches of the internal iliac arteries were of lower contrast resolution, making them less useful for guidance. The planning software in AS1 was also liver-specific, resulting in lower performances in pelvic anatomy segmentation compared with AS2 software, thus making the use of CBCT planning and augmented fluoroscopy more challenging in AS1. Additionally, a digital zoom function was available in AS2, allowing for the enlargement of fluoroscopic images without having to use true magnification, reducing the radiation dose due to fluoroscopy. Last but not least, significant design differences exist between different vendors’ imaging chains resulting in significant differences in dose rates in similar conditions.

In the selection of PAEs to be included in this study, a higher percentage of them were excluded from AS1 because they did not include a CBCT (see Figure 1). There are two factors to explain this. First, the distance from the source to the detector during CBCT was greater in AS2, making acquiring a CBCT possible for patients with a large body habitus. Second, in the authors’ experience, preparing the fluoroscope to acquire a CBCT, particularly centering the anatomy, was much more cumbersome and time-consuming in AS1. These are also the rationales for why the standard workflow in AS2 involved most typically two selective CBCT angiograms versus one less selective CBCT angiogram in AS1.

There are multiple limitations associated with this analysis, including the retrospective study design and non-randomized assignment of patients to the two angiography suites. Moreover, two different procedural imaging techniques were utilized, weakening the comparison. The conclusions would have been much stronger if the same technique was employed with both angiography systems and if the two techniques were compared on the same angiography system. Potential additional areas of bias include the use of a single operator and a non-randomized selection of the radial vs. femoral approach [6]. It should also be noted that operators naturally had more experience performing PAE by the time AS2 was implemented, which may account for some of the reduced radiation exposure. Because of the retrospective nature, there were missing data from both groups. These included data needed to assess what percentage of total procedural radiation doses were attributable to fluoroscopy, DSA and CBCT. Because of the potential importance of the missing BMI data resulting in bias, a multiple imputation sensitivity analysis was performed that supported the conclusions provided by the raw dataset. It is also important to reiterate that the AS2 group had a significantly lower BMI, but to account for this, a statistical analysis that adjusted the radiation doses for the BMI was utilized, resulting in the same conclusions provided by the raw data. Additionally, this analysis does not speak to the efficacy of PAEs performed on each system with each technique because clinical outcome data were not included. This type of analysis would be of interest before making any practice changes based on this report.

In conclusion, patient radiation from AS1 was similar to that reported in other PAE studies, but it was substantially lower in AS2. The angiography system used has a significant impact on the ability to leverage CBCT and on overall patient and thus staff radiation exposure during PAE.

## Figures and Tables

**Table 1:** Baseline demographics.

	AS1 (*n* = 25)	AS2 (*n* = 28)	*p*-Value
Age in years (mean ± SD)	69.9 ± 8.9	71.1 ± 9.1	0.44
Body mass index (mean ± SD)	29.4 ± 4.6	26.2 ± 3.7	0.027
Prostate volume (mL)	103.0 ± 46.1	130.1 ± 69.8	0.23

AS1 = angiography system 1; AS2 = angiography system 2; SD = standard deviation.

**Table 2:** Comparison of radiation dose factors and outcomes.

	AS1 (*n* = 25)	AS2 (*n* = 28)	*p*-Value
Fluoroscopy time (minutes), mean ± SD	37.3 ± 12.9	32.1 ± 11.8	0.13
Number of DSA series, mean ± SD	19.8 ± 5.9	8.0 ± 5.3	<0.001
Number of CBCTs, mean ± SD	1.3 ± 0.5	2.1 ± 0.4	<0.001
Mean reference air kerma per CBCT (mGy), mean ± SD	217.8 ± 33.4	45.8 ± 13.6	<0.0001
Cumulative air kerma (mGy), mean ± SD	2020.4 ± 1230.2	490.3 ± 50.5	<0.0001
Total kerma area product (Gy*cm^2^), mean ± SD	259.3 ± 115.5	72.7 ± 73.0	<0.0001

AS1 = angiography suite 1; AS2 = angiography suite 2; DSA = digital subtraction angiography; CBCT = cone beam computed tomography; SD = standard deviation.

**Table 3:** Comparison of adjusted primary radiation dose outcomes.

	AS1 (*n* = 25)	AS2 (*n* = 28)	*p*-Value
**Mean reference air kerma per CBCT (log mGy)**	5.3 (SE 0.04)	3.9 (SE 0.04)	<0.0001
**Cumulative air kerma (log mGy)**	7.2 (SE 0.1)	6.1 (SE 0.1)	<0.0001
Total kerma area product (log (Gy*cm^2^))	5.3 (SE 0.1)	4.5 (SE 0.1)	<0.0001

AS1 = angiography suite 1; AS2 = angiography suite 2; CBCT = cone beam computed tomography; SE = standard error.

## Data Availability

The original contributions presented in this study are included in the article. Further inquiries can be directed to the corresponding author.
